# Evolutionary Screening of *Lacticaseibacillus rhamnosus* MP108 for Freeze–Thaw Tolerance

**DOI:** 10.3390/microorganisms14061240

**Published:** 2026-05-31

**Authors:** Lina Pan, Jiaqi Wang, Wei Li, Cailing Chen, Yuguang Wang, Ruixia Gu, Hengxian Qu, Hongbo Zhou

**Affiliations:** 1 School of Minerals Processing and Bioengineering, Central South University, Changsha 410083, China; lina.pan@ausnutria.com (L.P.);; 2 Ausnutria Dairy (China) Co., Ltd., Changsha 410200, China; jiaqi.wang@ausnutria.com (J.W.); wei.li@ausnutria.com (W.L.); cailing.chen@ausnutria.com (C.C.); 3 School of Food Science and Engineering, Yangzhou University, Yangzhou 225127, China

**Keywords:** *Lacticaseibacillus rhamnosus*, adaptive evolution, freeze tolerance, stability, antioxidant capacity

## Abstract

Freeze-drying is the most commonly used method for preserving probiotics. The freeze tolerance of probiotics has a significant impact on both their survival rate and the expression of their functional properties. To enhance the freeze tolerance of probiotics, this study established an adaptive evolution protocol combining cold stress with repeated freeze–thaw cycles to screen for freeze–thaw-tolerant evolved strains of *Lacticaseibacillus rhamnosus* MP108. The safety, metabolic, and functional characteristics of these strains were then evaluated. The results showed that the combination of the 8 h cold stress treatment at 4 °C and nine cycles of freezing and thawing at −20 °C effectively enhanced the strain’s freeze tolerance, and the evolved strain L134 was successfully screened through adaptive evolution. Its freeze-dried survival rate and storage survival rate after 6 months of storage were both significantly higher than those of the parental strain (*p* < 0.05). Furthermore, it exhibited good passage stability. At the same time, the safety and acid-producing characteristics of L134 did not show significant changes compared to the parental strain. Furthermore, its tolerance to simulated gastric fluid, antibacterial activity, and antioxidant capacity were significantly enhanced (*p* < 0.05). In particular, compared to MP108, L134 exhibited significantly increased hydroxyl radical scavenging capacity as well as higher activities of the antioxidant enzymes SOD and CAT (*p* < 0.05); the improvement in its freeze tolerance may be related to this enhanced antioxidant capacity.

## 1. Introduction

Probiotics are live microorganisms that confer health benefits to the human body. They can regulate the balance of the gut microbiota and promote nutrient absorption, immune regulation, and the suppression of pathogenic bacteria [1]. With growing public interest in health, probiotics are widely used in foods, dietary supplements, and pharmaceuticals [2]. The viability and stability of probiotics are key factors in ensuring their efficacy [3]. In practical applications, maintaining the viability of probiotics during production, transportation, and storage has become an urgent issue to address.

Freeze-drying is a commonly used method for preserving probiotics. It offers advantages such as long shelf life and ease of transportation [4]. However, during this process, extreme temperature fluctuations and water removal often lead to the formation of intracellular ice crystals and changes in osmotic pressure. This can compromise the integrity and fluidity of the bacterial cell membrane, as well as the structure of sensitive proteins, resulting in impaired physiological function and even bacterial death [5]. Probiotics also face the challenge of oxidative stress during the freeze-drying process. This damage not only reduces the survival rate of probiotics but may also affect their functional performance in the gut. Therefore, the freeze tolerance of probiotics has become a current research focus.

The theory of adaptive evolution plays a crucial role in enhancing the freeze tolerance of probiotics. Subjecting probiotics to cold stress can induce adaptive evolution in strains, thereby improving their ability to withstand extreme environments [6,7]. Meanwhile, repeated freeze–thaw treatment is considered another more effective optimization strategy. By simulating extreme fluctuations in natural environments, this approach prompts probiotics to enhance their stress response capabilities. Repeated freeze–thaw cycles promote the further adaptation and repair of cell membranes and internal structures, thereby strengthening resistance to damage caused during the freeze-drying process [8]. In this study, unlike the freeze-drying protocols used in most studies, by combining these two strategies, it is expected that the freeze survival rate of probiotics can be improved while maintaining their metabolic activity and functional characteristics.

*Lacticaseibacillus rhamnosus* MP108 (MP108) was isolated from the intestines of healthy Chinese infants and young children. Its primary benefits include regulating the gut microbiota [9], treating ulcerative colitis [10], and alleviating hemolytic jaundice [11]. To enhance the freeze tolerance of MP108, this study employed an adaptive evolution approach, utilizing a combined strategy of cold stress and repeated freeze–thaw cycles to screen for freeze–thaw-tolerant evolved strains of MP108. The storage stability, safety, metabolic, and functional characteristics of the strain were evaluated both before and after modification. This not only provides a new research perspective for preserving high activity in freeze-dried probiotics—helping ensure that functional probiotics maintain high activity levels in products—but also offers a scientific basis for the development of highly effective and stable probiotic products.

## 2. Materials and Methods

### 2.1. Strain

*Lacticaseibacillus rhamnosus* MP108, sourced from Ausnutria Dairy (China) Co., Ltd. (Changsha, China).

### 2.2. Strain Activation

Glycerol tubes containing *Lacticaseibacillus rhamnosus* MP108 were removed from a −80 °C environment and placed in a 37 °C water bath. After complete thawing, a loop was used to inoculate de Man, Rogosa and Sharpe (MRS) (Hopebiol, Qingdao, China) agar plates for streaking. The plates were incubated upside down at 37 °C for 24–48 h, after which a single colony was picked with a loop and streaked onto a new plate. After performing streak plating three times, an inoculation loop was used to transfer a single colony to MRS liquid medium for further cultivation.

### 2.3. Screening for Adaptive Evolutionary Conditions

#### 2.3.1. Screening for Cold Stress Conditions

Using MP108 as the parental strain, a 2% inoculum of the activated bacterial suspension was taken during the mid-log phase (37 °C, 16 h). After adjusting the OD_600_ to 0.6, cold stress was applied. The stress system consisted of 1 mL. The grouping method is shown in Table 1. After cold stress treatment, repeated freeze–thaw cycles were performed using a standardized protocol. The bacterial suspension in each group was frozen at −80 °C for 24 h, then thawed in a 37 °C water bath for 5 min. This freeze–thaw cycle was repeated three times, followed by a viable cell count to compare the freeze–thaw survival rates for each group at each cycle.

#### 2.3.2. Screening of Freeze–Thaw Temperatures

After treatment under the optimal cold stress conditions, the suspension was subjected to three freeze–thaw cycles at −80 °C and −20 °C (freezing for 24 h at the respective temperatures, followed by a 5 min incubation in a 37 °C water bath). The control group underwent freeze–thaw cycles without prior cold stress. The pre-freezing temperature was selected by evaluating the freeze–thaw survival rate.

#### 2.3.3. Screening of Freeze–Thaw Cycles

After selecting the optimal cold stress conditions, the samples were subjected to the optimal freeze–thaw temperature cycle 3, 6, and 9 times. Live cell counts were performed after 0, 3, 6, and 9 freeze–thaw cycles. The control group consisted of samples subjected to direct freeze–thaw treatment, without cold stress. The number of freeze–thaw cycles was selected by evaluating the freeze–thaw survival rate of each group.

### 2.4. Screening for Freeze-Tolerant Strains

**Figure 1:**
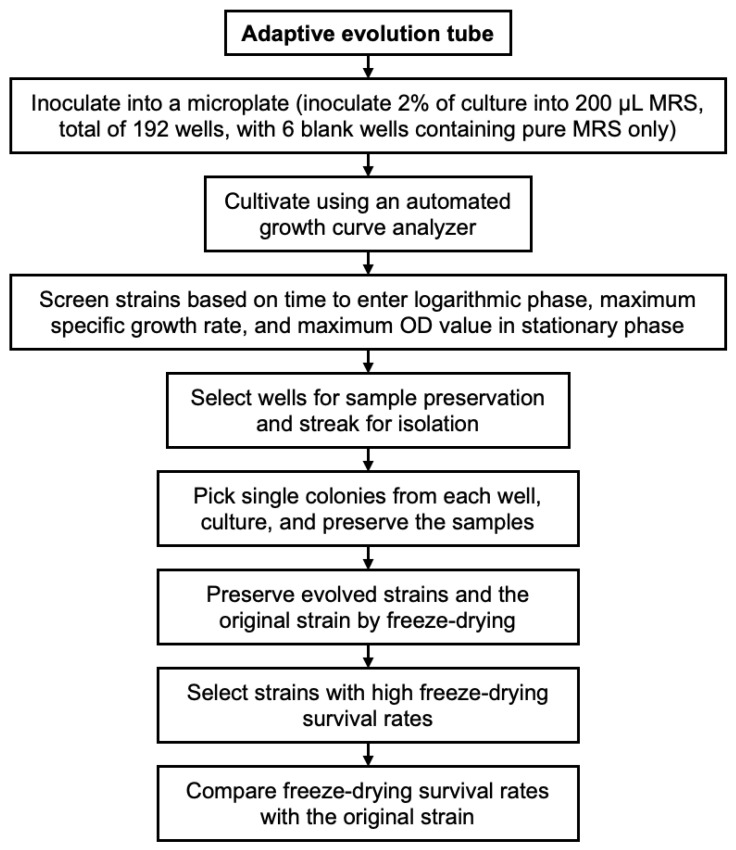
The process of adaptive evolution.

Based on the criteria established in the preliminary screening, the parent strain MP108 was subjected to cold stress. Following repeated freeze–thaw cycles, the strain was isolated and screened using a fully automated growth curve analyzer. The bacterial suspension was inoculated into two 96-well plates, with 200 μL of fresh medium added to each well. The freeze–thawed bacteria were inoculated at a 2% concentration, numbered from L1 to L180 in the order of administration, and six wells were set aside as medium blanks. The specific process is shown in Figure 1. Evolved strains were selected based on the growth rate and the speed at which they entered the logarithmic phase. After streaking and purifying the selected evolved strains, freeze-drying tests were conducted (freeze-drying temperature setting: pre-freezing at −45 °C for 4 h; −20 °C for 12 h; −1 °C for 12 h; 20 °C for 12 h; with physiological saline as the protective agent) to evaluate the freeze-drying survival rates of each evolved strain (equivalent-mass rehydration).

### 2.5. Passage Stability Assay

The evolved strain and the parental strain MP108 were passaged consecutively. Bacterial suspensions from the 1st, 5th, and 10th generations were selected; after centrifugation, a preservative (12% skim milk, 4% trehalose, 2% monosodium glutamate, and 4% glycerol) or saline solution was added to conduct freeze-drying survival rate verification experiments to assess the passage stability of the freeze-drying survival rate of the evolved strain.

### 2.6. Storage Stability Test

The evolved strain and the parental strain MP108 were lyophilized after the addition of a cryoprotectant and stored at −20 °C, 4 °C, and 25 °C. Their survival rates were compared over a 6-month storage period to evaluate the storage stability of the evolved strain.

### 2.7. Strain Safety Evaluation

The safety of the strains was evaluated through antibiotic susceptibility testing (Applicable Standards: CLSI M100-2026) and hemolysis assays.

### 2.8. Acid Production Characteristics of Strains

Following the method described in Reference [12], the concentrations of lactic acid, acetic acid, and propionic acid produced by the strains after 16 h of fermentation at 37 °C (late logarithmic phase) were determined.

### 2.9. Gastrointestinal Tolerance of Strains

MRS liquid medium containing 0.3% pepsin (Solarbio, Beijing, China) and with a pH of 3.0 was prepared to simulate artificial gastric fluid. MRS liquid medium containing 0.1% trypsin (Solarbio, Beijing, China) and 0.1% bovine bile salts (Solarbio, Beijing, China) was prepared to simulate artificial intestinal fluid. A total of 1.0 mL of bacterial suspension was inoculated into 9.0 mL of simulated gastric fluid and intestinal fluid and incubated anaerobically at 37 °C for 3 h. The number of viable bacteria at 0 h and 3 h was determined using the plate count method, and the survival rate (%) of the strain in the simulated gastric fluid and intestinal fluid was calculated according to Formula (1). Tolerance was compared by comparing survival rates.

Survival rate = Number of viable bacteria at 3 h/Number of viable bacteria at 0 h × 100%(1)

### 2.10. In Vitro Antimicrobial Activity of Strains

The evolved strain L134 and the starter strain MP108 were inoculated at a concentration of 2% into MRS liquid medium and fermented for 16 h to obtain the fermentation broth. The antimicrobial activity of the strains against *Bacillus subtilis* ATCC 6633, *Bacillus cereus* ATCC 14579, *Escherichia coli* ATCC 25922, *Staphylococcus aureus* ATCC 6538, and *Salmonella typhimurium* ATCC14028 was assessed using the agar diffusion method. A total of 200 μL of overnight bacterial culture was spread evenly onto solidified LB agar. Next, a 6 mm diameter well was punched into the plate using a sterile punch, and 100 μL of the bacterial culture was inoculated into the well. The plates containing the samples were incubated at 37 °C for 24 h. The diameter of the inhibition zone was measured three times using a Vernier caliper (Mitutoyo, Shanghai, China). Antibacterial activity was compared by measuring the size of the inhibition zones.

### 2.11. In Vitro Antioxidant Activity of Strain

The evolution strain L134 and the parental strain MP108 were inoculated at a concentration of 2% into MRS liquid medium and fermented for 16 h to obtain the fermentation broth. Meanwhile, the cells were collected by centrifugation, washed twice with saline, resuspended, and adjusted to an OD_600_ of 0.6 to obtain the cell sample. Following the instructions in the kit, the antioxidant capacity of the bacterial culture supernatant and the bacterial cells was determined using the DPPH Radical Scavenging Assay Kit, Hydroxyl Radical Assay Kit, and Total Antioxidant Capacity (T-AOC) Assay Kit (Solarbio, China). The antioxidant enzyme activities in the bacterial culture supernatant (MRS medium) were determined using the Superoxide Dismutase (SOD) Activity Assay Kit, Catalase (CAT) Activity Assay Kit, and Reduced Glutathione (GSH) Content Assay Kit (Solarbio, China).

### 2.12. Data Analysis

Statistical analysis was performed using GraphPad Prism 11 (San Diego, CA, USA). All experiments were conducted with three experimental replicates and three technical replicates. The results are presented as the means ± SDs, and the differences among the different samples were analyzed using a one-way analysis of variance (ANOVA, Tukey). Values of *p* < 0.05 were considered statistically significant.

## 3. Results and Discussion

### 3.1. Screening of Conditions for Adaptive Evolution

**Figure 2:**
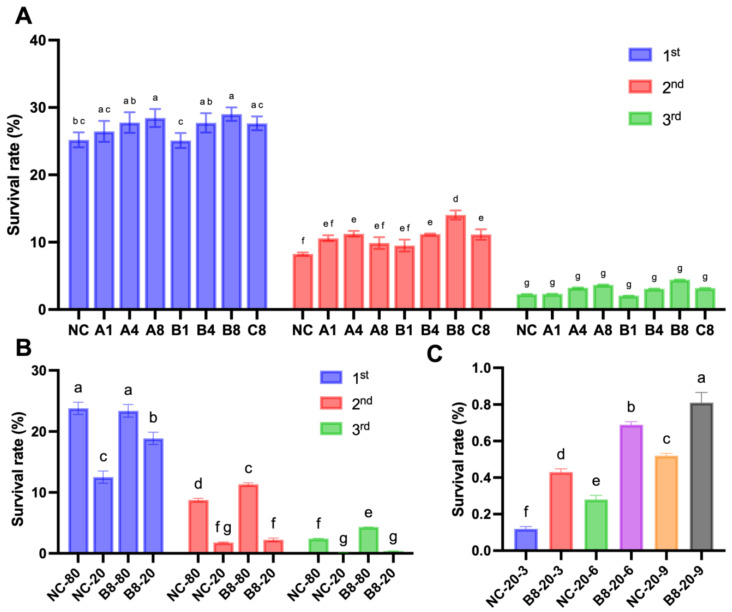
Screening of adaptive evolution conditions. (**A**) Effect of cold stress on strain survival rate. (**B**) Effect of pre-freezing temperature on strain survival rate. (**C**) Effect of freeze–thaw cycles on strain survival rate. In (**B**,**C**), suffixes 20 and 80 denote freeze–thaw cycles at −20 °C and −80 °C, respectively, while suffixes 3, 6, and 9 represent number of freeze–thaw cycles. Different letters indicate significant differences (*p* < 0.05).

The conditions for the adaptive evolution of strain MP108 were screened by evaluating the effects of different cold stress and freeze–thaw conditions on the strain’s survival rate. The results are shown in Figure 2.

As shown in Figure 2A, after the first freeze–thaw cycle, Group B8 exhibited the highest bacterial survival rate, which was significantly higher than that of Groups NC and B1 (*p* < 0.05). After the second freeze–thaw cycle, the survival rate of Group B8 was significantly higher than that of the other groups (*p* < 0.05). After the third freeze–thaw cycle, the survival rate of Group B8 remained higher than that of the other groups, although the difference was not statistically significant. The fact that Group B8 exhibited a higher survival rate after all three freeze–thaw cycles indicates that this treatment helps enhance the strain’s freeze tolerance. Cold stress prompts bacteria to adjust the fatty acid composition of their cell membrane phospholipid bilayer, increasing the proportion of unsaturated fatty acids. Unsaturated fatty acids have a lower melting point and can maintain cell membrane fluidity at low temperatures, thereby better protecting against membrane damage caused by ice crystal formation and osmotic pressure changes during the freeze-drying process [13]. Maintaining this membrane fluidity is crucial for preserving cell integrity during freeze-drying and rehydration. Consequently, in this study, the survival rates of strains subjected to cold stress showed varying degrees of improvement after freeze–thaw cycles compared to those in the untreated group.

As shown in Figure 2B, a comparative study of freeze–thaw cycles following cold stress revealed that, compared to the control group, the survival rate was lower in the −20 °C freeze–thaw group than in the −80 °C freeze–thaw group. Different freezing temperatures affect the size of ice crystals formed inside and outside bacterial cells during freezing, thereby influencing the extent of physical damage to the cells. Yang [14] also found that, compared to slow freezing at −20 °C, the rapid freezing of *Lactiplantibacillus plantarum* LIP-1 at −80 °C significantly improved survival rates during freeze-drying. Since pre-freezing at −20 °C causes greater damage to the strains, only those with stronger freeze resistance survive, which facilitates the screening and isolation of freeze-tolerant strains.

As shown in Figure 2C, a comparative study of the number of freeze–thaw cycles following cold stress revealed that after nine freeze–thaw cycles, the freeze–thaw survival rates of both the non-cold-stressed group and the cold-stressed group increased significantly (*p* < 0.05), indicating that repeated freeze–thaw cycles enhance the strain’s freeze tolerance. Research by Sooyeon Song [15] also showed that after *Lactobacillus plantarum* L67 underwent cold stress involving four consecutive freeze–thaw cycles, its frost resistance improved and freeze-drying survival rate increased. Furthermore, a study on *Lactobacillus rhamnosus* GG subjected to 150 freeze–thaw–growth cycles found that the evolved mutant strain exhibited mutations at multiple genetic loci. These genes are involved in membrane lipid, cell wall, and extracellular polysaccharide synthesis, driving multi-target genetic changes that led to favorable alterations in the physical properties of the overall cellular structure, thereby comprehensively enhancing stress tolerance [8]. These findings, together with those of this study, confirm that repeated freeze–thaw cycles can enhance the freeze tolerance of lactic acid bacteria. While some adaptive evolution studies employ a large number of repeated cycles, the defining feature of adaptive evolution is not the cycle count per se but the demonstration of heritable phenotypic gains under a defined selective regime. In the present study, despite the relatively modest number of nine freeze–thaw cycles, the evolved populations exhibited significantly improved survival and stress tolerance, which constitutes evidence of adaptive evolution under combined cold and freeze–thaw pressure.

Based on the above, wild-type strains were subjected to a 4 °C, 8 h cold stress treatment and nine cycles of freezing and thawing at −20 °C for the screening of freeze–thaw-tolerant strains.

### 3.2. Isolation and Screening of Freeze-Dried-Tolerant Strains

**Figure 3:**
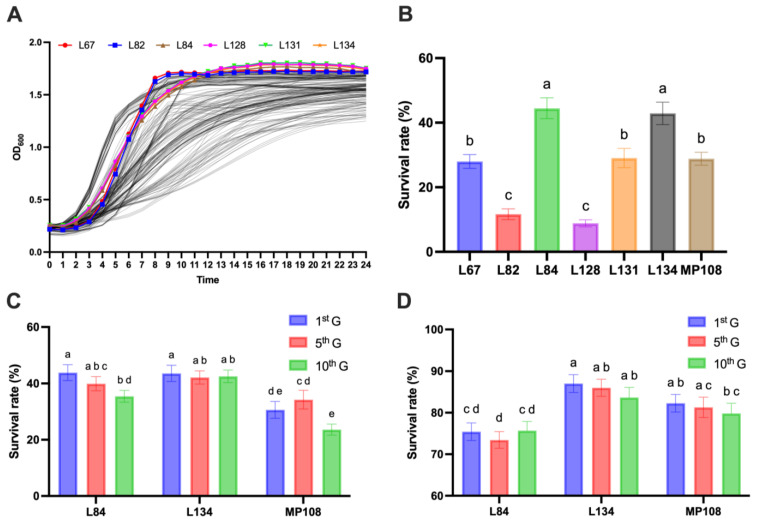
Isolation and screening of freeze-dried-tolerant strains. (**A**) Growth curves of screened strains. (**B**) Freeze-drying survival rates of screened strains. (**C**) Freeze-drying survival rates of screened strains at different passage numbers. (**D**) Freeze-drying survival rates of screened strains after addition of protective agent. Different letters indicate significant differences (*p* < 0.05).

After subjecting the parental strain MP108 to cold stress (4 °C for 8 h), it was subjected to nine cycles of freeze–thawing at −20 °C. Screening was performed using a fully automated growth curve analyzer, and the freeze-dried survival rates and passage stability of the strains were compared. The results are shown in Figure 3.

As shown in Figure 3A, a total of 180 wells were inoculated. Based on the growth rates and logarithmic phase durations of each strain, superior wells were screened by selecting those with the highest growth rates, higher OD values during the stationary phase, and faster entry into the logarithmic phase. Strains L67, L82, L84, L128, L131, and L134 and the parental strain were selected for subsequent freeze-drying survival rate experiments. A comparison of their freeze-drying survival rates revealed that, as shown in Figure 3B, the freeze-drying survival rates of strains L84 and L134 were significantly higher than that of the parental strain (*p* < 0.05).

The passage stability of probiotics is of paramount importance. A genetically stable strain maintains a stable genome during long-term passaging and large-scale production, without undergoing changes that could affect its core characteristics. To obtain genetically stable evolved strains, strains L87 and L134 were selected for a comparison of freeze-dried survival rates after passaging. As shown in Figure 3C, L87, L134, and MP108 all maintained good passage stability after five passages, with no significant differences in their freeze-dried survival rates. However, after 10 passages, only L134 showed no significant difference in the freeze-dried survival rate compared to the first-generation strain. This indicates that L134 possesses better passage stability. As shown in Figure 3D, L134 still exhibited good stability after the addition of a cryoprotectant. Even after 10 passages, the freeze-drying survival rate of L134 remained statistically indistinguishable from that of the first-generation strain. Although L84 also demonstrated good stability in the presence of the cryoprotectant, its freeze-drying survival rate was significantly lower than that of L134 (*p* < 0.05).

After cold stress and repeated freeze–thaw cycles, the screened L134 strain exhibited the highest freeze tolerance, with a freeze-dried survival rate significantly higher than that of MP108. Furthermore, the freeze-dried survival rate of the L134 strain remained stable after continuous passaging, indicating good passage stability. Therefore, L134 was selected as the freeze-tolerant adaptive evolution strain of MP108. This is also consistent with the theory of probiotic adaptive evolution, which posits that environmental stress can induce beneficial mutations in strains, thereby enhancing their adaptability.

### 3.3. Storage Stability of Evolved Strains

**Figure 4:**
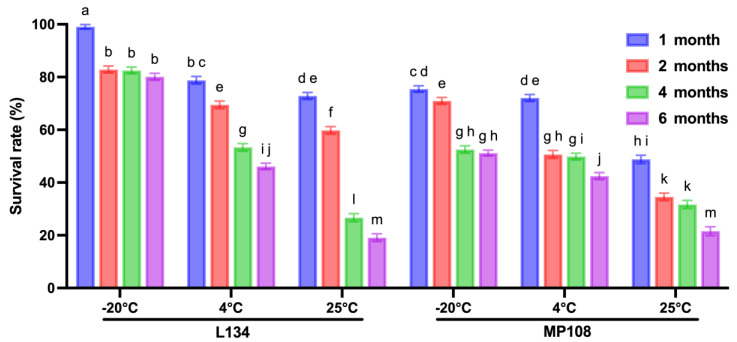
Storage stability of evolved strains. Different letters indicate significant differences (*p* < 0.05).

The survival rates of freeze-dried bacterial powders from the evolved strains L134 and MP108 were investigated at different storage temperatures; the results are shown in Figure 4.

As shown in Figure 4, the storage stability of L134 and MP108 decreases as the temperature rises. The strain L134 maintained a high survival rate (over 80%) even after 6 months of storage at −20 °C. Furthermore, its survival rate was significantly higher than that of MP108 throughout the storage period (*p* < 0.05), indicating better storage stability. However, no significant differences in storage stability were observed under storage conditions at 4 °C and 25 °C. The evolutionary strain L134, selected through adaptive evolution, exhibits enhanced tolerance in multiple aspects. This improvement is not limited to withstanding the freeze-drying process but also enhances its overall ability to cope with oxidative stress and other challenges during long-term storage. The cell membranes of the evolved strain have undergone multiple rounds of stress screening, resulting in optimized fatty acid composition, membrane proteins, and lipid structure, thereby conferring higher intrinsic stability. This not only provides protection during the freeze-drying process but also enables the strain to better resist membrane lipid peroxidation and degradation caused by oxidation and dehydration during long-term storage. Consequently, the evolved strain L134 maintains a higher survival rate during storage at −20 °C. In addition, the improvement in the storage stability of L134 is temperature-dependent; high temperatures have a significant impact on survival rates, resulting in no difference in the final survival rates of L134 and MP108.

### 3.4. Safety Evaluation of Evolved Strains

**Figure 5:**
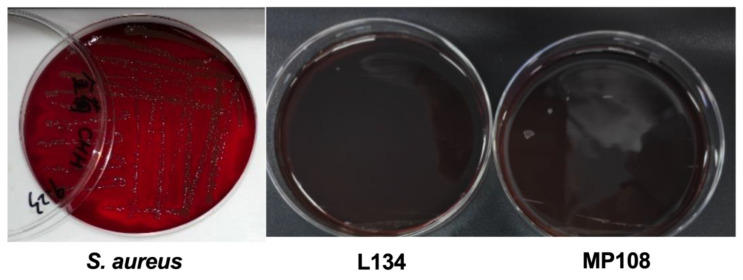
Hemolytic activity of evolved strains.

The antibiotic susceptibility and hemolytic activity of the evolved strains L134 and MP108 were determined; the results are shown in Table 2 and Figure 5.

During adaptive evolution, probiotics may upregulate their inherent resistance mechanisms in response to stress while simultaneously losing some functional genes, thereby affecting the safety of the strains. Therefore, a safety assessment of evolved strains is of critical importance. MP108 has been previously evaluated for safety in accordance. In the present study, MP108 was not intended as a novel strain requiring de novo safety evaluation but rather served as a control to enable a comparative assessment of potential safety-related changes in the evolved L134 strain. As shown in Table 2, the parental strain MP108 and the evolved strain L134 are resistant to norfloxacin and cefradine and are not susceptible to them; they are susceptible to clarithromycin. Compared with the parental strain MP108, the evolved strain L134 is more susceptible to rifampin, indicating that the evolved strain still possesses good safety. Additionally, the hemolytic activity of typical bacterial strains is classified as α-hemolysis (formation of a grass-green hemolytic zone), β-hemolysis (formation of a clear hemolytic zone), and γ-hemolysis (no hemolytic zone). If no hemolytic zone appears around the colony, it indicates that the bacterium lacks hemolytic capacity and is not a potential pathogen. As shown in Figure 5, neither the parental strain nor the modified strain exhibits a hemolytic zone, further confirming their safety.

### 3.5. Study on Acid Production Characteristics of Evolved Strains

**Figure 6:**
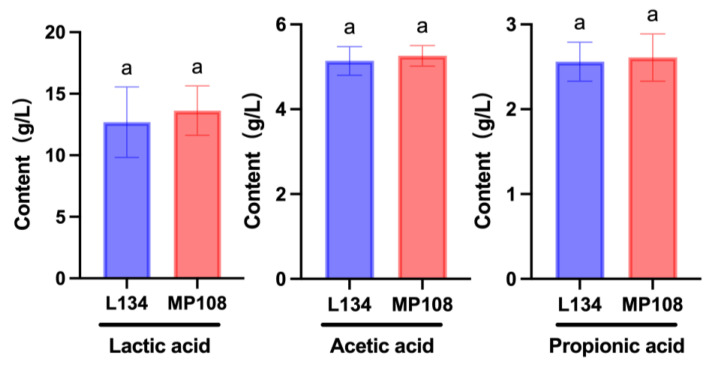
Acid-producing properties of evolved strains. Different letters indicate significant differences (*p* < 0.05).

By analyzing the levels of organic acids (lactic acid, acetic acid, and propionic acid) in the fermentation broths of evolved strains L134 and MP108, the acid production characteristics of the two strains were compared. The results are shown in Figure 6.

As shown in Figure 6, both the parental strain MP108 and the evolved strain L134 produced lactic acid, acetic acid, and propionic acid during fermentation at 37 °C, and there were no significant differences in the yields of these three organic acids. This indicates that the optimization for cold tolerance did not affect the strain’s key metabolic pathways and, therefore, did not affect its acid production characteristics. A study on the continuous acid stress adaptive evolution of *Bifidobacterium longum* BBMN68 also found that genomic changes in the mutant strain were primarily concentrated in genes related to extracellular polysaccharide synthesis, while genes in key metabolic pathways remained unchanged [16]. This may be the reason why its acid production characteristics were preserved.

### 3.6. Study on Gastrointestinal Tolerance of Evolved Strains

**Figure 7:**
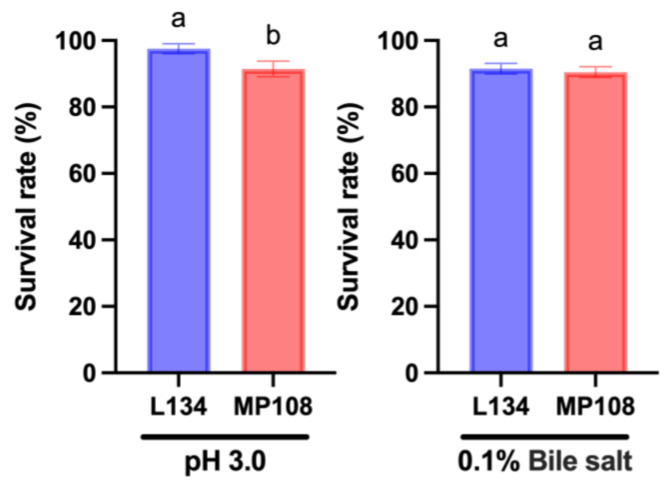
Gastrointestinal tolerance of evolved strains. Different letters indicate significant differences (*p* < 0.05).

The acid and bile salt tolerance of the evolved strains L134 and MP108 were investigated, and the results are shown in Figure 7.

Only a small number of probiotics with good acid tolerance are able to survive the gastric acid barrier and reach the gastrointestinal tract to exert their effects. As shown in Figure 7, compared to the parental strain MP108, the evolved strain L134 exhibits greater tolerance to a pH 3.0 environment, with a significantly higher survival rate (*p* < 0.05). There was no significant difference in bile salt tolerance between the parental strain MP108 and the evolved strain L134. The cell membrane serves as the first line of defense in maintaining intracellular pH homeostasis. An intact, stable membrane structure can more effectively prevent the influx of protons, thereby protecting the cell interior from acid damage. Both low-temperature freezing and simulated gastrointestinal stress can cause severe damage to the cell membrane. During the process of adaptive evolution, the composition of L134’s cell membrane may have changed, thereby helping it adapt to freezing stress. This change in cell membrane composition also contributes to enhanced resistance to acid stress. But it should still be noted that although the observed increase in the survival rate reached statistical significance, the magnitude of the difference between the evolved and parental strains was moderate.

### 3.7. Study of Antimicrobial Activity of Evolved Strains

**Figure 8:**
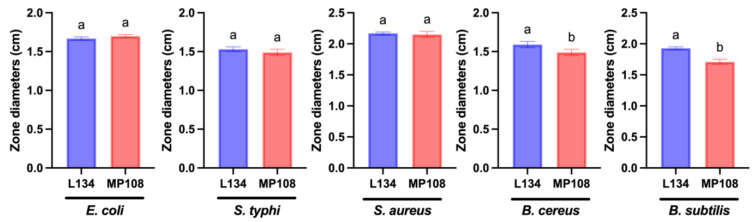
Antimicrobial activity of evolved strains. Different letters indicate significant differences (*p* < 0.05).

The antimicrobial activity of the evolved strains L134 and MP108 was investigated, and the results are shown in Figure 8.

As shown in Figure 8, the inhibitory activity of the evolved strain L134 against *B. cereus* and *B. subtilis* was significantly higher than that of MP108, while there were no significant changes in its inhibitory activity against *E. coli*, *S. typhi*, and *S. aureus*. The metabolic antibacterial profile of the evolved strain L134 underwent specific changes, exhibiting enhanced selective inhibitory activity against the two Bacillus species. Although acid production is the primary mechanism of antibacterial activity in probiotics, the acid-producing capacity of the evolved strain L134 did not show significant changes compared to the parental strain MP108. However, the strain L134, following adaptive evolution, still exhibited changes in the metabolism of other metabolites related to antibacterial activity. Interestingly, this new combination of metabolites may be particularly effective in inhibiting the growth of *B. cereus* and *B. subtilis* while showing no effect on other bacterial species, a finding that warrants further investigation.

### 3.8. Study on Antioxidant Capacity of Evolved Strains

**Figure 9:**
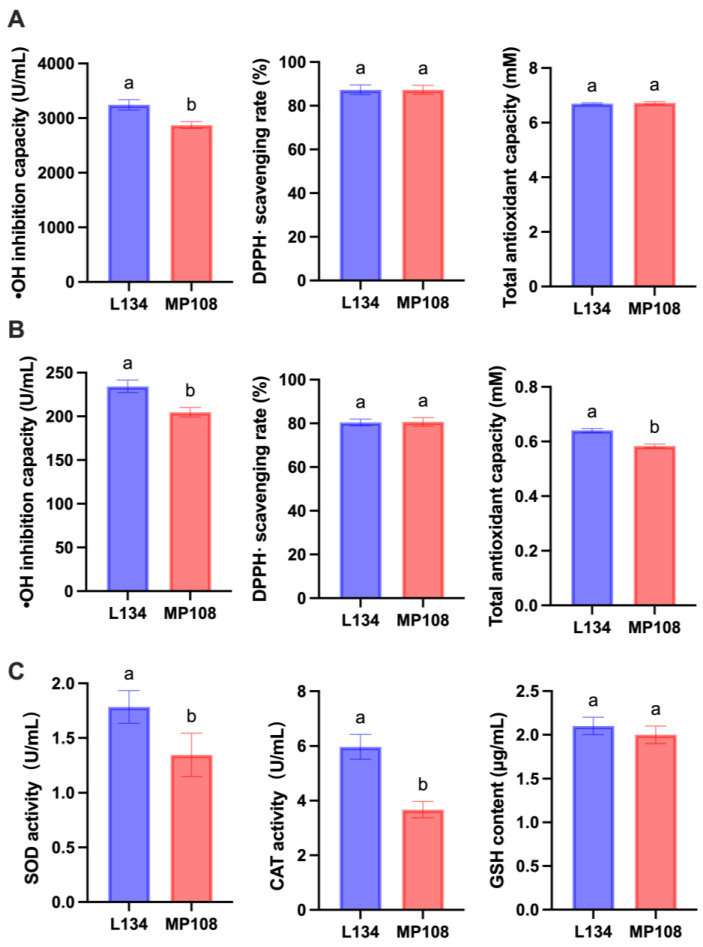
Antioxidant capacity of evolved strains. (**A**) Antioxidant capacity of fermentation broth. (**B**) Antioxidant capacity of bacterial cells. (**C**) Antioxidant enzyme activity. Different letters indicate significant differences (*p* < 0.05).

The antioxidant capacity of the evolved strains L134 and MP108 was investigated, and the results are shown in Figure 9.

As shown in Figure 9, compared with MP108, both the fermentation broth and the bacterial cells of L134 exhibited significantly enhanced hydroxyl radical scavenging capacity (*p* < 0.05), and the total antioxidant capacity of L134 bacterial cells was also significantly increased (*p* < 0.05). Furthermore, the activities of the antioxidant enzymes SOD and CAT in the L134 fermentation broth were both significantly elevated (*p* < 0.05). The process of adaptive evolution selects mutant strains capable of continuously synthesizing or accumulating more endogenous protective substances [17]. There is a close physiological link between cold stress and oxidative stress [18]. Evolutionary pressure enhances the strain’s overall antioxidant defense system, including the activity of enzymes such as superoxide dismutase (SOD) and catalase (CAT) [17]. This was also verified in the present study: following adaptive evolution, both the antioxidant capacity and the activity of antioxidant enzymes in L134 were significantly enhanced. These enzyme systems effectively scavenge reactive oxygen species (ROS) generated during freeze-drying and storage, reducing oxidative damage. Consequently, while improving freeze-drying survival rates, they also significantly enhanced the strain’s stability and survival rates during storage. Studies have shown that in strains that have acquired high tolerance through adaptive evolution, the enhancement in tolerance is often closely related to the improvement in enzymatic antioxidant capacity [19]. This suggests that increasing SOD and CAT activity is an effective strategy for enhancing the tolerance of strains during the process of adaptive evolution.

## 4. Conclusions

In this study, we applied adaptive evolution combining cold stress and repeated freeze–thaw cycles to improve the stress tolerance of probiotic strain L134. The novelty of this approach lies in its use of multidimensional selective pressure (cold plus freeze–thaw) rather than a single stressor, which may better mimic the real-world challenges encountered during probiotic processing and storage.

We successfully optimized the freeze tolerance of *Lacticaseibacillus rhamnosus* through adaptive evolution strategies, identifying strain L134, which exhibits high freeze tolerance, good passage stability and in vitro probiotic properties. Furthermore, we found that the enhanced antioxidant capacity of probiotics during adaptive evolution is key to their improved tolerance. At the same time, several findings point to the influence of changes in aspects such as the composition of the bacterial cell membrane.

However, the causal relationship between enhanced antioxidant capacity and improved cold tolerance, as well as the specific mechanisms by which changes in cell membrane composition affect cold damage, remain speculative. Furthermore, the stability of the evolved strains requires further validation, indicating certain limitations. This warrants further in-depth research to elucidate the mechanisms by which the evolved strain resists freeze damage. Future studies should address these limitations by integrating multi-omics approaches, molecular characterization, in vivo testing, and longer-term passage stability assessments.

## Figures and Tables

**Table 1:** Adaptation treatment methods.

Group	Treatment Method
NC	Subject the aliquoted bacterial suspension to repeated freeze–thaw cycles directly, without prior cold acclimation.
A1	Place the bacterial suspension at 8 °C for 1 h, and then subject it to repeated freeze–thaw cycles.
A4	Place the bacterial suspension at 8 °C for 4 h, and then subject it to repeated freeze–thaw cycles.
A8	Place the bacterial suspension at 8 °C for 8 h, and then subject it to repeated freeze–thaw cycles.
B1	Place the bacterial suspension at 4 °C for 1 h, and then subject it to repeated freeze–thaw cycles.
B4	Place the bacterial suspension at 4 °C for 4 h, and then subject it to repeated freeze–thaw cycles.
B8	Place the bacterial suspension at 4 °C for 8 h, and then subject it to repeated freeze–thaw cycles.
C8	Implement a stepwise cooling process: allow the aliquots of bacterial suspension to stand at room temperature (20 °C) for 1 h, and then lower the temperature by 2 °C every hour until it reaches 4 °C. After a total cooling period of 8 h, perform repeated freeze–thaw cycles.

**Table 2:** Antibiotic susceptibility testing.

Antibiotic	Inhibition Zone Diameter (mm)		Susceptibility	
	L134	MP108	L134	MP108
Rifampin	24.67 ± 0.58 ^a^	17.67 ± 0.58 ^b^	S	I
Clarithromycin	28.33 ± 1.53 ^a^	23.33 ± 1.53 ^b^	S	S
Cefadroxil	-	-	R	R
Norfloxacin	-	-	R	R

Notes: S, Susceptible; I, Intermediate; R, Resistant. Different letters indicate significant differences (*p* < 0.05).

## Data Availability

The data presented in this study are available on request from the corresponding author. The data are not publicly available due to privacy restrictions.
